# Intraperitoneal versus intranasal administration of lipopolysaccharide in causing sepsis severity in a murine model: a preliminary comparison

**DOI:** 10.1186/s42826-024-00205-7

**Published:** 2024-05-13

**Authors:** Yaqing Jiao, Cindy S. W. Tong, Lingyun Zhao, Yilin Zhang, John M. Nicholls, Timothy H. Rainer

**Affiliations:** 1 Department of Emergency Medicine, School of Clinical Medicine, Li Ka Shing Faculty of Medicine, The University of Hong Kong, Hong Kong, China; 2 Department of Pathology, School of Clinical Medicine, Li Ka Shing Faculty of Medicine, The University of Hong Kong, Hong Kong, China

**Keywords:** LPS, Intraperitoneal, Intranasal, Sepsis, Organ injury, Histology

## Abstract

Community-acquired respiratory infection is the commonest cause of sepsis presenting to emergency departments. Yet current experimental animal models simulate peritoneal sepsis with intraperitoneal (*I.P.*) injection of lipopolysaccharide (LPS) as the predominant route. We aimed to compare the progression of organ injury between *I.P.* LPS and intranasal (*I.N.*) LPS in order to establish a better endotoxemia murine model of respiratory sepsis. Eight weeks old male BALB/c mice received LPS-*Escherichia coli* doses at 0.15, 1, 10, 20, 40 and 100 mg per kg body weight (e.g. LPS-10 is a dose of 10 mg/kg body weight). Disease severity was monitored by a modified Mouse Clinical Assessment Score for Sepsis (M-CASS; range 0–21). A M-CASS score ≥ 10 or a weight reduction of ≥ 20%, was used as a criterion for euthanasia. The primary outcome was the survival rate (either no death or no need for euthanasia). The progression of disease was specified as M-CASS, body weight, blood glucose, histopathological changes to lung, liver, spleen, kidney, brain and heart tissues. Survival rate in *I.P.* LPS-20 mice was 0% (2/3 died; 1/3 euthanized with M-CASS > 10) at 24 h. Survival rate in all doses of *I.N.* LPS was 100% (20/20; 3–4 per group) at 96 h. 24 h mean M-CASS post-*I.P.* LPS-10 was 6.4/21 significantly higher than *I.N.* LPS-10 of 1.7/21 (Unpaired t test, *P* < 0.05). Organ injury was present at 96 h in the *I.P.* LPS-10 group: lung (3/3; 100%), spleen (3/3; 100%) and liver (1/3; 33%). At 24 h in the *I.P.* LPS-20 group, kidney injury was observed in the euthanized mouse. At 96 h in the post-*I.N.* LPS-20 group, only lung injury was observed in 2/3 (67%) mice (Kruskal-Wallis test with Dunn’s, *P* < 0.01). At 24 h in the post-*I.N.* LPS-100 group all (4/4) mice had evidence of lung injury. Variable doses of *I.N.* LPS in mice produced lung injury but did not produce sepsis. Higher doses of *I.P.* LPS induced multi-organ injury but not respiratory sepsis. Lethal models of respiratory virus, e.g., influenza A, might provide alternative avenues that can be explored in future research.

**Supplementary Information:**

The online version contains supplementary material available at 10.1186/s42826-024-00205-7.

## Background

Sepsis is a life-threatening failure of body organs caused by a dysregulated host-response to infection [1]. Community-acquired respiratory infection is the commonest cause of sepsis presenting to emergency departments. Respiratory sepsis is defined as sepsis caused by respiratory infections.

Murine models of sepsis have been developed in an effort to create controllable and reproducible, experimental systems for investigating mechanisms of sepsis development and for studying therapeutics [2]. An injection of lipopolysaccharide (LPS) is one common, low-invasive model to provide certain features of the acute state of sepsis [3]. It is highly suitable for investigating acute inflammation, although this model does not involve true pathogen infections [4]. LPS derives from the outer membrane of Gram-negative bacteria. High levels of LPS in the bloodstream are observed in bacteremia which may progress to life-threatening sepsis [5]. LPS, as a relatively pure chemical, can be quantitatively measured, and its administration can be standardized [2, 3].

Intraperitoneal (*I.P.*) administration is predominantly used to construct LPS models. Intranasal (*I.N.*) administration of LPS is rarely used but has been reported to induce persistent rhinitis in mouse models [6, 7]. *I.N.* administration of live pathogens is widely used to construct pneumonia models [3, 8]. There is no evidence comparing the disease progression between *I.P.* and *I.N.* administration of LPS. No studies evaluate the comparison of multi-organ injury between *I.P.* and *I.N.* LPS. We postulate that the *I.N.* LPS model better represents organ injury due to respiratory sepsis than the *I.P.* model. In this study, we compared the two routes of administration in constructing an LPS-induced inflammation model. We reported the differences between *I.N.* LPS and *I.P.* LPS in inducing the clinical course and progression to organ injury in murine models.

## Main text

This study was conducted under the license of the Committee on the Use of Live Animals in Teaching and Research (CULATR approval number: 5924-21). This study was carried out by persons holding valid Cap. 340 licenses issued by the Department of Health, and the principles of laboratory animal care were followed along the study. Eight-week old male BALB/c mice (body weight 25–30 g) were used, as the workflow shown in Additional file 1. LPS at 0.15, 1, 10 and 20 mg/kg body weight for both *I.P.* and *I.N.* routes, and 40 and 100 mg/kg for *I.N.* route only, were tested. Animals were monitored for 4 days using a modified Mouse Clinical Assessment Score for Sepsis (M-CASS) based on appearance (fur aspect), activity, posture, response to stimulus, chest movements, chest sounds and eye lids (Additional file 2) [9, 10]. Mice that appeared to be suffering (a total score reaching 10) or that had a weight reduction of ≥ 20%, were deemed unhealthy and an early scarification was carried out. A score of 10 or a weight loss of 20% was allocated to dead mice to facilitate subsequent statistical analysis. There were three or four mice in each group. At humane endpoints, 24 h and 96 h, mice were sacrificed to collect brain, heart, lung, liver, spleen and kidney tissues. Tissues were fixed in formalin, trimmed and dehydrated, and cut into 6 μm thin sections stained by hematoxylin and eosin (H&E). Five microscopy photos for each tissue each mouse at the field of 20 × were taken, and analyzed semi-quantitatively (Additional file 3).

All analyses and figures were computed with the program GraphPad Prism (v.9 GraphPad Software, USA). In all cases, data were produced from 3 to 4 animals. Statistical significance was determined by Kruskal-Wallis test, Mann-Whitney U test and t test. *P* < 0.05 was considered as statistically significant; *P* < 0.01, *P* < 0.001 and *P* < 0.0001 were further specified. Data were presented as median with 95% confidence interval (CI), median with 2.5–97.5 percentile and mean with standard deviation (SD).

**Fig. 1:**
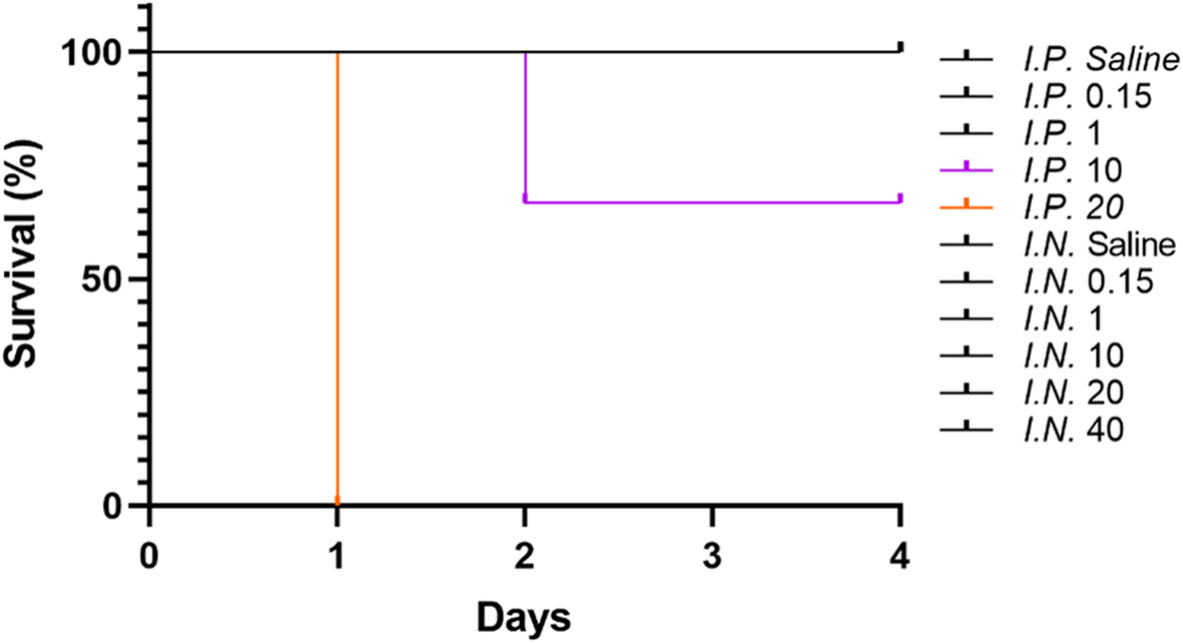
Survival curve of 8-week-old male Balb/c mice after treated with different LPS doses at 0.15, 1, 10 and 20 mg/kg body weight via intraperitoneal (*I.P.*) route, and 0.15, 1, 10, 20 and 40 mg/kg body weight via intranasal (*I.N.*) route over 96 h. Three or four mice for each group

In the *I.P.* groups, 8-week-old male BALB/c mice were injected with 0.15, 1, 10 or 20 mg/kg body weight of LPS and monitored for 96 h. After treatment with 10 mg/kg LPS, the survival rate was 66.7%; and after treatment with 20 mg/kg LPS, the survival rate dropped to 0% (Fig. 1). By comparison, no mice died in the *I.N.* groups, even when being treated with 40 mg/kg LPS (Fig. 1).

**Fig. 2:**
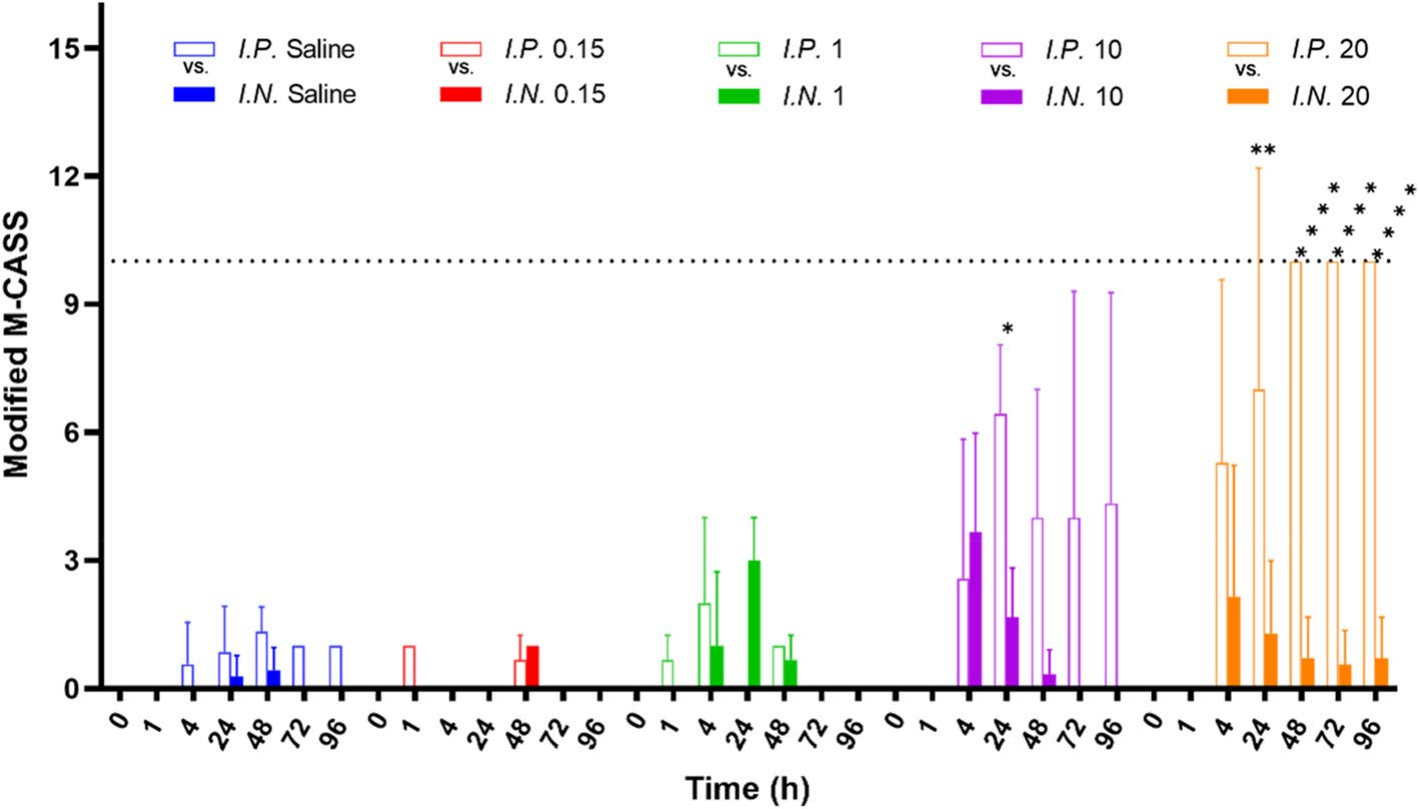
Measurement of a modified Mouse Clinical Assessment Score for Sepsis (M-CASS) of 8-week-old male Balb/c mice after intraperitoneal (I.P.) or intranasal (I.N.) treatment with 0.9% saline, 0.15 mg/kg LPS, 1 mg/kg LPS, 10 mg/kg LPS and 20 mg/kg LPS over 96 h. Data were presented as mean with standard deviation (SD). Comparison of M-CASS between I.P. LPS and I.N. LPS at the same dose was made by unpaired t test with Welch correction (* *P* < 0.05; ** *P* < 0.01; **** *P* < 0.0001). Three or four mice for each group. Note: A total score of 10 or more, or when one category reaches a 3, was used as a criterion for euthanasia; as for the dead mice, a score of 10 was allocated at the time points after mice died

At 4 h, mice started to exhibit abnormal symptoms in both routes (Fig. 2). The symptoms were more severe at higher doses, suggesting a dose-response relationship. *I.P.* LPS induced relatively higher scores than *I.N.* counterparts, aligning with the survival rate. At 24 h, mean M-CASS post-*I.P.* LPS-10 was 6.4/21 significantly higher than *I.N.* LPS-10 of 1.7/21 (*P* < 0.05; Fig. 2). The conditions worsened the most at 24 h after which mice either died or recovered.

**Fig. 3:**
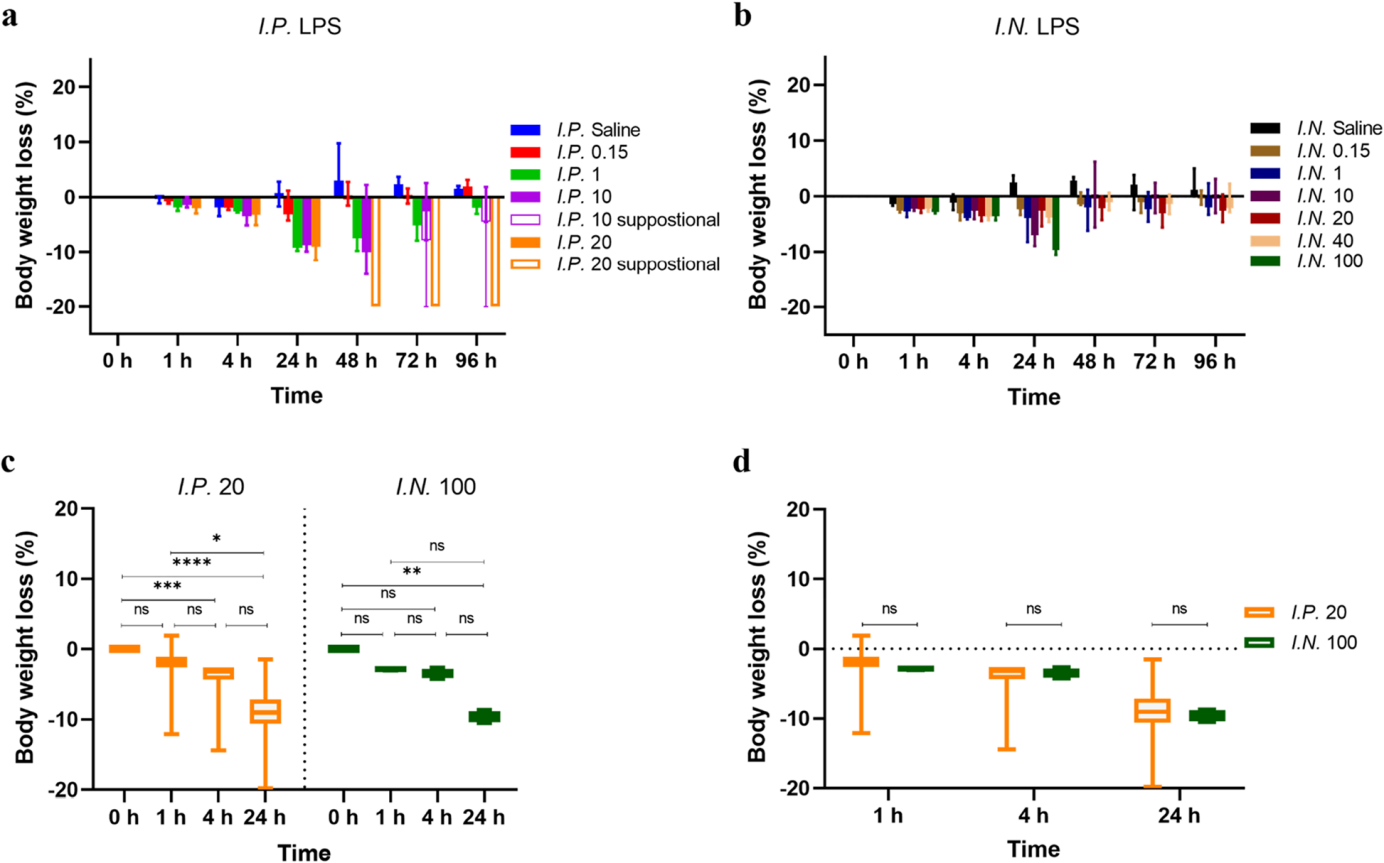
Measurement of body weight loss (%) of 8-week-old male Balb/c mice after intraperitoneal (*I.P.*) treatment with 0.15, 1, 10 and 20 mg/kg LPS over 96 h (**a**), measurement of body weight loss (%) of mice after intranasal (*I.N.*) treatment with 0.15, 1, 10, 20 and 40 mg/kg LPS over 96 h, and 100 mg/kg LPS over 24 h (**b**), comparison of body weight loss (%) of mice between 0 h, 1 h, 4 h and 24 h for *I.P. *20 mg/kg LPS stimulation and *I.N.* 100 mg/kg LPS stimulation, respectively (Kruskal-Wallis test with Dunn’s multiple comparisons test; ns = not significant; * *P* < 0.05; ** *P* < 0.01; *** *P* < 0.001; **** *P* < 0.0001) (**c**), comparison of body weight loss (%) of mice between *I.P.* 20 mg/kg LPS stimulation and *I.N.* 100 mg/kg LPS stimulation at 1 h, 4 h and 24 h (Mann-Whitney test with correction for multiple comparisons using the Holm-Sidak method) (**d**). Data were presented as median with 95% confidence interval (CI) in Fig. 4a and b, and median with 2.5-97.5 percentile in Fig. 4c and d. Three or four mice for each group. Note: suppositional = A body weight loss of 20 % which has been defined as one humane endpoint was allocated at the time points after mice died

**Fig. 4:**
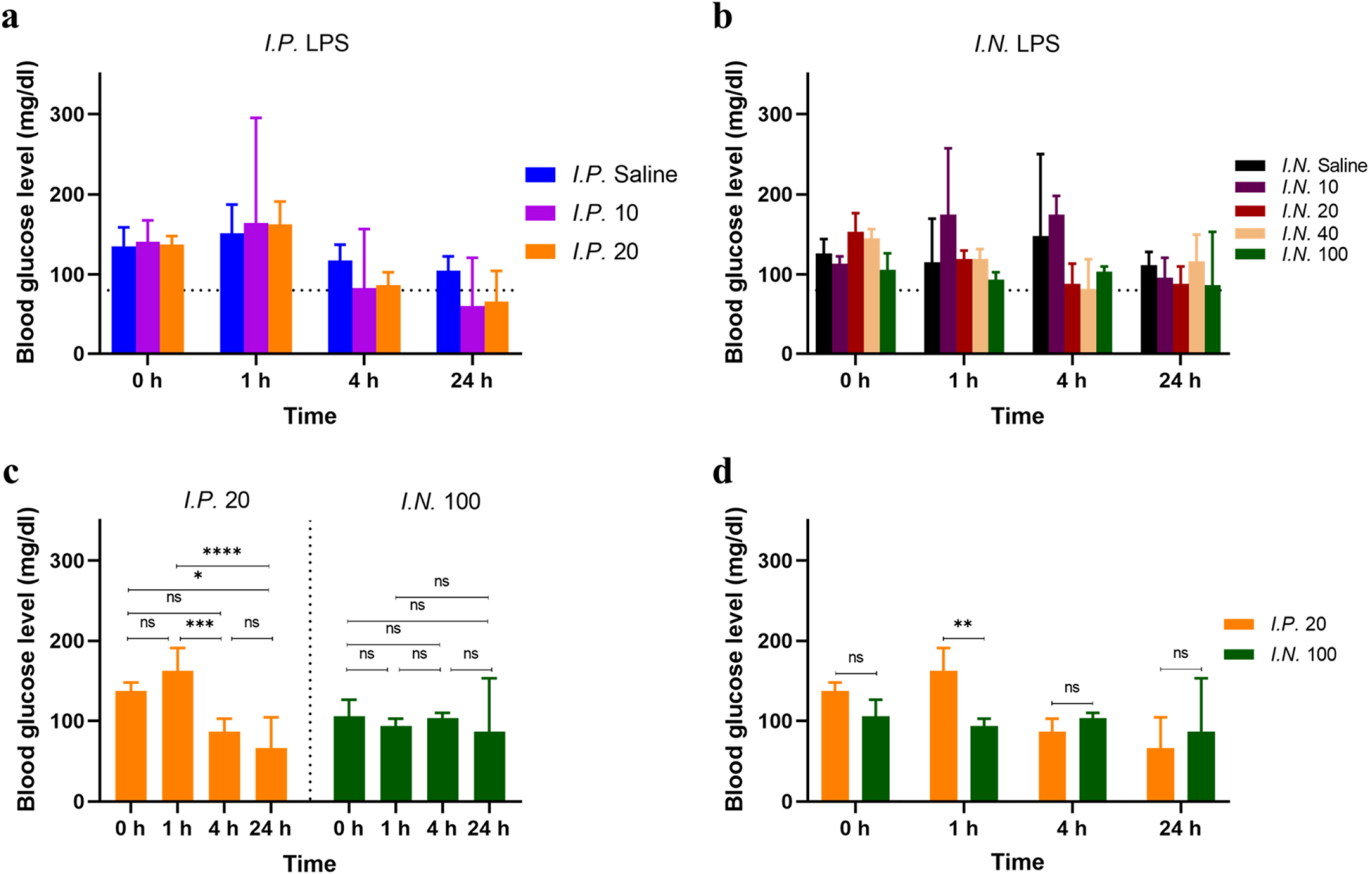
Measurement of blood glucose level (mg/dl) of 8-week-old male Balb/c mice after intraperitoneal (*I.P.) *treatment with 10 mg/kg LPS, 20 mg/kg LPS and 0.9% saline over 24 h (**a**), measurement of blood glucose level (mg/dl) of mice after intranasal (*I.N.*) treatment with 10 mg/kg LPS, 20 mg/kg LPS, 40 mg/kg LPS, 100 mg/kg LPS and 0.9% saline over 24 h (**b**), comparison of blood glucose level (mg/dl) of mice between 0 h, 1 h, 4 h and 24 h for *I.P.* 20 mg/kg LPS stimulation and *I.N. *100 mg/kg LPS stimulation, respectively (Kruskal-Wallis test with Dunn’s multiple comparisons test; ns = not significant; * *P* < 0.05; ** *P* < 0.01; *** *P* < 0.001; **** *P* < 0.0001) (**c**), comparison of blood glucose level (mg/dl) of mice between *I.P. *20 mg/kg LPS stimulation and *I.N. *100 mg/kg LPS stimulation at 1 h, 4 h and 24 h (Mann-Whitney test with correction for multiple comparisons using the Holm-Sidak method) (**d**). Data were presented as median with 95% confidence interval (CI). Three or four mice for each group. Note: for the groups in which mice died, blood glucose level of the whole group has been removed from analysis thereafter

Body weight dropped the most at 24 h and then recovered in survival mice (Figs. 3a and 4b). In the *I.P.* groups, 1 mg/kg or higher doses induced a 10% weight loss within 24 h, and the loss became significant at 4 h and 24 h (*P* < 0.001; Fig. 3c). In the *I.N.* groups, only the mice treated with 100 mg/kg LPS dropped 10% weight at 24 h and only this drop was significant (*P* < 0.01; Fig. 3c). The trend in weight loss was similar between the two routes (*P* > 0.05; Fig. 3d).

**Fig. 5:**
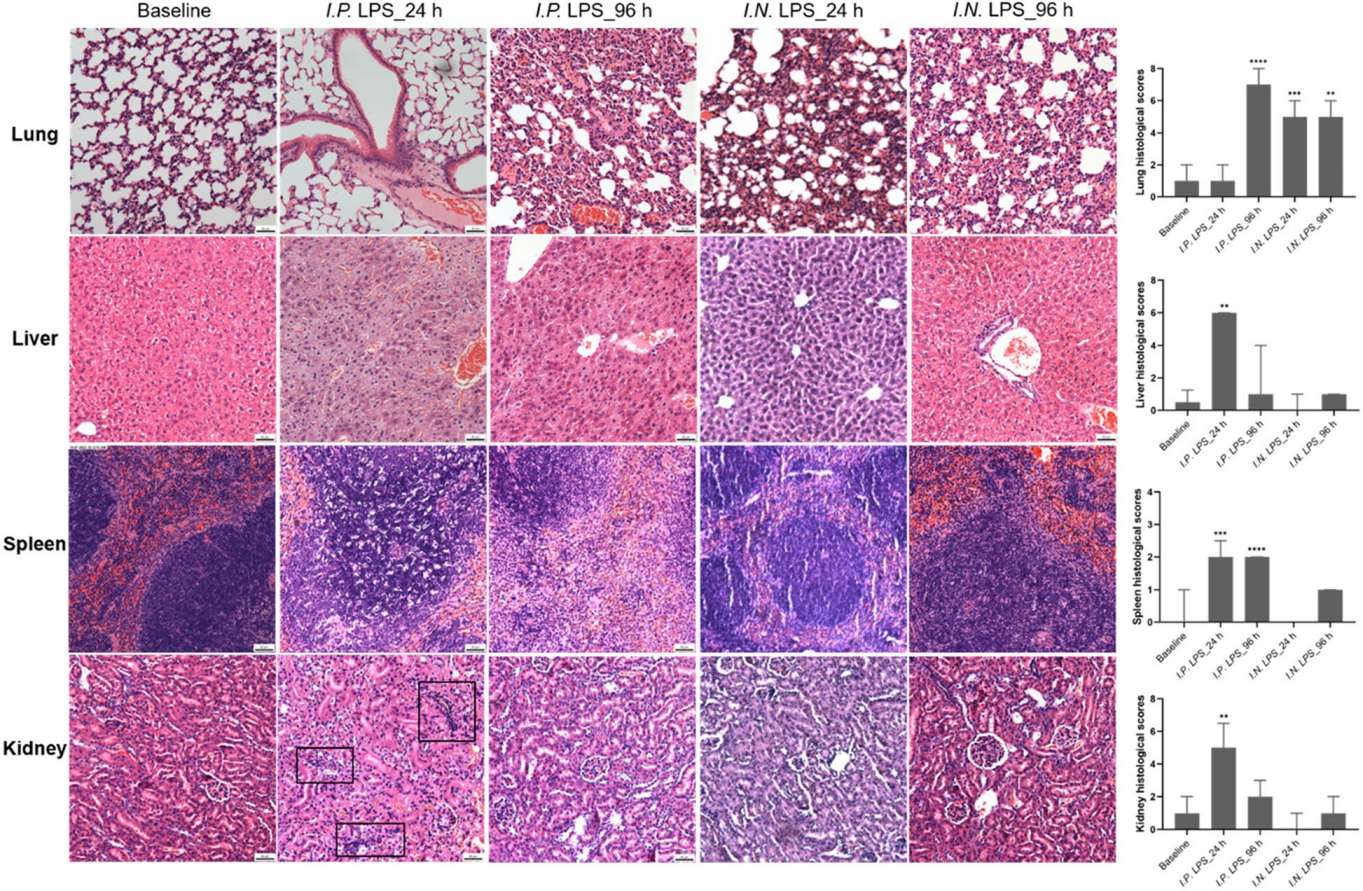
Histology of lung, liver, spleen and kidney from control and lipopolysaccharide (LPS) treated mice via intraperitoneal (*I.P.*) or intranasal (*I.N.*) routes at 24 h and 96 h stained with hematoxylin and eosin (H&E). Size bar = 50 µm. Histological scores were achieved by semi-quantitative scoring systems (Additional file 2), and statistical analysis was performed by Kruskal-Wallis test with Dunn’s multiple comparison test (ns = not significant; * *P*< 0.05; ** *P* < 0.01; *** *P* < 0.001; **** *P*< 0.0001; data were presented as median with interquartile; three or four mice for each group). Note: Sample availability was affected by the death of mice and/or experimental design. Representative graphs from one of 3-4 mice in each group are shown

Blood glucose levels were measured using a commercial kit of blood glucose meter (Safe-Accu, Sinocare, China). Blood glucose levels fluctuate in patients with sepsis. Hyperglycemia and hypoglycemia can develop, affecting health outcomes and reflecting the severity of sepsis. A fasting blood glucose level of 80–100 mg/dL has been reported for healthy, nondiabetic mice [11, 12]. In the *I.P.* groups, the glucose level of mice treated with 10 and 20 mg/kg LPS both decreased below 80 mg/dl at 4 h and 24 h, indicating hypoglycemia (Figs. 4a and 5c). The blood glucose change was not LPS dose-dependent (Figs. 4a and 5b). At 1 h, a single rising glucose level was recorded, inferring a rapid, sharper anti-stress reaction through intensive energy consumption to LPS stimulation (Figs. 4a and 5b). In the *I.N.* groups, the glucose level was relatively stable (Figs. 4b and 5d).

Lung tissues of the baseline and *I.P.* LPS mice at 24 h presented normal structure with thin classical inter-alveolar septa, clear bronchioles (BR) and vessels (Fig. 5). Lung tissues of the *I.P.* LPS mice at 96 h and the *I.N.* LPS mice at both 24 h and 96 h were observed with thickened alveolar septa, reduced alveolar spaces, massive cellular infiltration in alveolar septa and collapsed alveoli (*P* < 0.01; Fig. 5). Therefore, *I.P.* LPS may induce lung injury at late stages, while *I.N.* LPS may induce lung injury rapidly. In the liver, only *I.P.* LPS mice at 24 h exhibited hepatocytic vacuolation and diffusing hemorrhage, with an injury score significantly higher than the baseline (*P* < 0.01; Fig. 5). Normal spleen tissues showed a compact structure of white pulps (WP) which is clearly distinguished from the surrounding red pulps (RP). An expansion of WP and widespread blank spots in WP nodules appeared in the *I.P.* LPS mice; and the boundary between WP and RP was blurring (Fig. 5). Mild disorder of WP in the *I.N.* LPS mice at 96 h was observed but is not significant (Fig. 5). In the kidney, only *I.P.* LPS mice at 24 h exhibited pathological structure featured with hemorrhage and infiltration of inflammatory cells (Fig. 5). In the brain and heart, similar structures nearly as the baseline were observed in all groups (Additional file 4). In the saline control, no pathological changes were observed (Additional file 5). Overall, *I.P.* LPS showed evidence of tissue damage on multiple organs, and *I.N.* LPS only produced a focused lung injury.

Most cases of sepsis are due to community-acquired respiratory infections acquired oro-nasally. As the *I.P.* route produces an intra-abdominal rather than respiratory, model of sepsis, it is less likely to be more relevant to community-acquired pneumonia. As *I.N.* is administration via the nose, it should be more physiologically relevant to respiratory sepsis. In this study, we compared the progression of organ injury between *I.P.* LPS and *I.N.* LPS in order to establish a better endotoxemia murine model of respiratory sepsis. Intratracheal (*I.T.*) and intrabronchial (*I.B.*) administration are also used to construct models of respiratory infections but these high-invasive routes require anesthesia which may interfere with immune responses [3, 13].

In our study, *I.P.* and *I.N.* LPS at the same doses induced strikingly different survival rates. *I.P.* LPS was more lethal than *I.N.* LPS, which may be due to differences between abdominal and pulmonary immunity. Mucosal surfaces, such as respiratory epithelium, are directly exposed to the external environment and therefore, are highly vulnerable to infection [14]. Environmental pathogens invade hosts most commonly through the olfactory mucosa [6, 7]. As a result, the respiratory tract has evolved a variety of innate and adaptive immune defenses, together with mucociliary clearance, to prevent infection, promote rapid destruction of infected cells and clear the invasions [14, 15]. Some vaccines can easily provoke robust systemic immune responses but relatively poor mucosal responses in the respiratory tract [14]. At the same dose, *I.N.* LPS might get compromised by the mucosal protection on the respiratory tract to elicit less harm than *I.P.* LPS. Unlike live pathogens, LPS as bacterial components are unable to grow and reproduce in hosts. Therefore, LPS via *I.N.* route may be unable to induce sepsis in mice.

Due to the death/humane endpoint of mice, the availability of tissue samples was affected. All the mice of *I.P.* LPS-20 died after 24 h. Therefore, at 96 h, tissues from *I.P.* LPS-10 survival mice were used to demonstrate histology alterations. Only *I.N.* route received higher LPS doses of 40 mg/kg (tested 96 h) and 100 mg/kg (tested 24 h). For M-CASS, *I.P.* and *I.N.* LPS at the same doses were compared. For body weight and glucose level, the highest doses from each route were chosen to represent the comparison.

The administration route of LPS plays an essential role in inducing acute pulmonary damage [16]. In a previous study, LPS administered *I.N.* caused acute pulmonary damage (within 2–4 h and reached maximal damage at 24–48 h), whereas intravenous (*I.V.*) LPS only produced a baseline effect [16]. However, that study did not investigate the injury of multiple organs. Our study revealed a consistent massive cellular infiltration in alveolar septa in lung tissues. Our study also revealed that *I.N.* LPS is unable to develop a similar degree of multi-organ (lung, liver, spleen and kidney) injury as with *I.P.* LPS. Nevertheless, great heterogeneity has been stated as the typical pattern of histopathological analysis in sepsis models. Organ injury was not always consistently observed in septic mice. No specific or characteristic histological features exist among different studies [9, 17].

We applied M-CASS to monitor disease severity. In humans, the Sequential Organ Failure Assessment (SOFA; additional file 6) score is widely used to assess the severity of sepsis [18]. Similar metrics to evaluate animal sepsis are lacking [9]. Only a limited amount of blood can be sampled in mice, rendering difficulty in performing biochemistry assays such as the measurement of platelets, bilirubin and creatinine [9]. M-CASS and the murine sepsis score (MSS) are commonly used to assess the severity of sepsis in rats and mice [9]. These tools are based on observational characteristics lacking the quantitative strength and relevance of SOFA. In future studies, we propose to develop a murine-SOFA to more objectively assess sepsis in murine models. Murine models infected with viruses have been widely studied, but few have been assessed from the perspective of sepsis. We suspect lethal virus models probably develop sepsis before death. In future research, lethal models of respiratory virus, e.g., influenza A, might provide alternative avenues that can be explored.

## Conclusions

In conclusion, variable doses of *I.N.* LPS in mice did not produce sepsis, as determined by M-CASS and histology. *I.P.* higher doses of LPS induced multi-organ injury but not respiratory sepsis. Lethal models of respiratory virus hold the potential to be explored in future research.

### Supplementary Information

Additional file 1. The workflow of comparing intraperitoneal (*I.P.*) and intranasal (*I.N.*) administration of lipopolysaccharide (LPS) in causing sepsis severity in a murine model.

Additional file 2. A modified Mouse Clinical Assessment Score for Sepsis (M-CASS).

Additional file 3. Semi-quantitative scoring systems for histological analysis.

Additional file 4. Histology of brain and heart from control and lipopolysaccharide (LPS) treated mice via intraperitoneal (*I.P.* 20_24 h & 10_96 h) or intranasal (*I.N.* 100_24 h & 20_96 h) routes at 24 h and 96 h stained with hematoxylin and eosin (H&E). Size bar = 50 μm.

Additional file 5. Histology of lung, liver, spleen, kidney, brain and heart from 0.9% saline treated mice via intraperitoneal (*I.P.*) or intranasal (*I.N.*) routes at 96 h stained with haematoxylin and eosin (H&E). Size bar = 50 μm.

Additional file 6. Sequential Organ Failure Assessment (SOFA) score.

## Data Availability

All datasets, on which the conclusions of the manuscript rely on, are presented in the paper.

## Abbreviations

BR: Bronchioles
CI: Confidence interval
H&E: Haematoxylin and eosin
I.B.: Intrabronchial
I.N.: Intranasal
I.P.: Intraperitoneal
I.T.: Intratracheal
I.V.: Intravenous
LPS: Lipopolysaccharide
M-CASS: Mouse Clinical Assessment Score for Sepsis
MSS: Murine Sepsis Score
RP: Red pulps
SD: Standard deviation
SOFA: Sequential Organ Failure Assessment
WP: White pulps

## References

[1] Singer M, Deutschman CS, Seymour CW, Shankar-Hari M, Annane D, Bauer M (2016). The third international consensus definitions for sepsis and septic shock (Sepsis-3). JAMA.

[2] Lewis AJ, Seymour CW, Rosengart MR (2016). Current murine models of sepsis. Surg Infect (Larchmt).

[3] Korneev KV (2019). Mouse models of Sepsis and Septic Shock. Mol Biol (Mosk).

[4] Seemann S, Zohles F, Lupp A (2017). Comprehensive comparison of three different animal models for systemic inflammation. J Biomed Sci.

[5] Kubicek-Sutherland JZ, Vu DM, Noormohamed A, Mendez HM, Stromberg LR, Pedersen CA (2019). Direct detection of bacteremia by exploiting host-pathogen interactions of lipoteichoic acid and lipopolysaccharide. Sci Rep.

[6] Casadei E, Salinas I (2019). Comparative models for human nasal infections and immunity. Dev Comp Immunol.

[7] Hasegawa-Ishii S, Shimada A, Imamura F (2017). Lipopolysaccharide-initiated persistent rhinitis causes gliosis and synaptic loss in the olfactory bulb. Sci Rep.

[8] Bhargava R, Altmann CJ, Andres-Hernando A, Webb RG, Okamura K, Yang Y (2013). Acute lung injury and acute kidney injury are established by four hours in experimental sepsis and are improved with pre, but not post, sepsis administration of TNF-α antibodies. PLoS ONE.

[9] Shrum B, Anantha RV, Xu SX, Donnelly M, Haeryfar SM, McCormick JK (2014). A robust scoring system to evaluate sepsis severity in an animal model. BMC Res Notes.

[10] Sulzbacher MM, Sulzbacher LM, Passos FR, Bilibio BLE, de Oliveira K, Althaus WF (2022). Adapted murine Sepsis score: improving the Research in Experimental Sepsis Mouse Model. Biomed Res Int.

[11] Wuyts C, Simoens C, Pinto S, Philippaert K, Vennekens R (2021). Continuous glucose monitoring during pregnancy in healthy mice. Sci Rep.

[12] Daniell H, Singh R, Mangu V, Nair SK, Wakade G, Balashova N (2023). Affordable oral proinsulin bioencapsulated in plant cells regulates blood sugar levels similar to natural insulin. Biomaterials.

[13] Müller-Redetzky H, Suttorp N, Witzenrath M (2012). Experimental models of pneumonia-induced sepsis. Drug Discovery Today: Disease Models.

[14] Allie SR, Randall TD (2017). Pulmonary immunity to viruses. Clin Sci (Lond).

[15] Eccles R (2021). The role of nasal congestion as a defence against respiratory viruses. Clin Otolaryngol.

[16] Szarka RJ, Wang N, Gordon L, Nation P, Smith RH (1997). A murine model of pulmonary damage induced by lipopolysaccharide via intranasal instillation. J Immunol Methods.

[17] Langenberg C, Bagshaw SM, May CN, Bellomo R (2008). The histopathology of septic acute kidney injury: a systematic review. Crit Care.

[18] Vincent JL, Moreno R, Takala J, Willatts S, De Mendonca A, Bruining H (1996). The SOFA (Sepsis-related Organ failure Assessment) score to describe organ dysfunction/failure. On behalf of the Working Group on Sepsis-related problems of the European Society of Intensive Care Medicine. Intensive Care Med.

