# Supplementary material for: Intraperitoneal versus intranasal administration of lipopolysaccharide in causing sepsis severity in a murine model: a preliminary comparison
Source: Lab Anim Res. 2024 May 13;40:18. doi: 10.1186/s42826-024-00205-7 (PMC11089766; doi:10.1186/s42826-024-00205-7)
Supplement: Supplementary file 1 — Additional file 1. The workflow of comparing intraperitoneal (I.P.) and intranasal (I.N.) administration of lipopolysaccharide (LPS) in causing sepsis severity in a murine model. [file 42826_2024_205_MOESM1_ESM.docx]

**
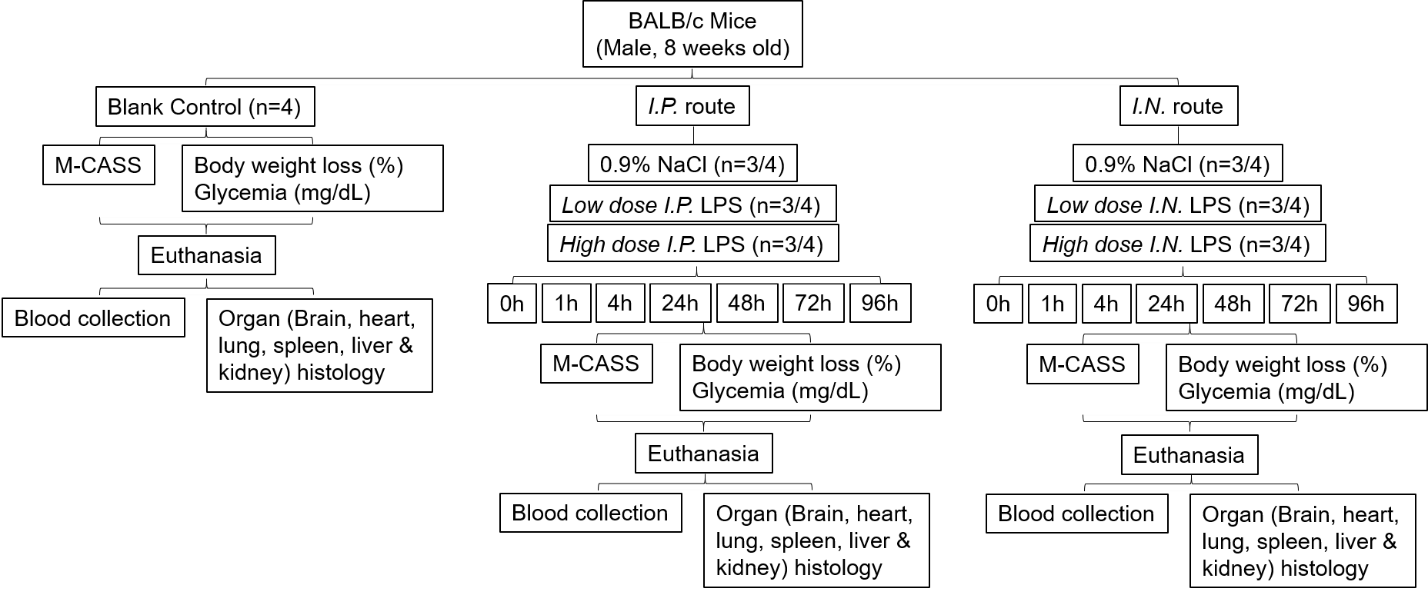
**

**Additional file 1** The workflow of comparing intraperitoneal (*I.P.*) and intranasal (*I.N.*) administration of lipopolysaccharide (LPS) in causing sepsis severity in a murine model.
