# Supplementary material for: Intraperitoneal versus intranasal administration of lipopolysaccharide in causing sepsis severity in a murine model: a preliminary comparison
Source: Lab Anim Res. 2024 May 13;40:18. doi: 10.1186/s42826-024-00205-7 (PMC11089766; doi:10.1186/s42826-024-00205-7)
Supplement: Supplementary file 2 — Additional file 2. A modified Mouse Clinical Assessment Score for Sepsis (M-CASS). [file 42826_2024_205_MOESM2_ESM.docx]

**Additional file 2** A modified Mouse Clinical Assessment Score for Sepsis (M-CASS).

| **Score** | **0** | **1** | **2** | **3** |
| --- | --- | --- | --- | --- |
| **Appearance (Fur aspect)** | Actively grooming | Dulling of hair coat | Rough hair coat | Piloerection |
| **Activity** | Normal activity | Reduced activity | No activity disturbed, reduced activity stimulated | No activity disturbed or stimulated |
| **Posture** | Normal | Slightly hunched, moving freely | Hunched with stiff movement / posture | Hunched with no movement stimulated |
| **Behavior** | Normal | Slow normal when disturbed | Abnormal disturbed, relocates only when stimulated | Abnormal when disturbed or stimulated, no relocation |
| **Chest movements** | Normal | Mildly dyspneic | Moderately dyspneic | Severely dyspneic with thoracic abdominal respiration |
| **Chest sounds** | No | Occasional chirping | Frequent chirping | Wet chirping increased when stimulated |
| **Eye lids** | Normally opened spontaneously | Normally opened disturbed | Near closed when stimulated and disturbed | Closed disturbed, near closed stimulated |
