# Supplementary material for: Intraperitoneal versus intranasal administration of lipopolysaccharide in causing sepsis severity in a murine model: a preliminary comparison
Source: Lab Anim Res. 2024 May 13;40:18. doi: 10.1186/s42826-024-00205-7 (PMC11089766; doi:10.1186/s42826-024-00205-7)
Supplement: Supplementary file 3 — Additional file 3. Semi-quantitative scoring systems for histological analysis. [file 42826_2024_205_MOESM3_ESM.docx]

**Additional file 3 Semi-quantitative scoring systems for histological analysis.**

| **Lung** | None  = 0 | | Minimal  = 1 | | Mild  = 2 | | Significant  = 3 | | Severe  = 4 | |
| --- | --- | --- | --- | --- | --- | --- | --- | --- | --- | --- |
| 1. Edema in the alveolar spaces |  | |  | |  | |  | |  | |
| 2. Infiltration of inflammatory cells |  | |  | |  | |  | |  | |
| 3. Hemorrhage |  | |  | |  | |  | |  | |
| 4. Thickness of the alveolar wall |  | |  | |  | |  | |  | |
| Sum score |  | | | | | | | | | |
|  |  |  |  |  |  |  |  |  |  |  |
| **Liver** | None  = 0 | | Minimal  = 1 | | Mild  = 2 | | Significant  = 3 | | Severe  = 4 | |
| 1. Destruction of hepatic lobules |  | |  | |  | |  | |  | |
| 2. Infiltration of inflammatory cells |  | |  | |  | |  | |  | |
| 3. Hemorrhage |  | |  | |  | |  | |  | |
| 4. Hepatocyte necrosis |  | |  | |  | |  | |  | |
| Sum score |  | | | | | | | | | |
|  |  | | | | | | | | | |
| **Kidney** | None  = 0 | | Mild  = 1 | | Moderate  = 2 | | Strong  = 3 | | Intense  = 4 | |
| 1. Degeneration |  | |  | |  | |  | |  | |
| 2. Necrosis |  | |  | |  | |  | |  | |
| 3. Infiltration of inflammatory cells |  | |  | |  | |  | |  | |
| 4. Hemorrhage |  | |  | |  | |  | |  | |
| Sum score |  | | | | | | | | | |
|  |  |  |  |  |  |  |  |  |  |  |
| **Brain** | None  = 0 | | Slight  = 1 | | Mild  = 2 | | Moderate  = 3 | | Severe  = 4 | |
| 1. Degeneration |  | |  | |  | |  | |  | |
| 2. Edema |  | |  | |  | |  | |  | |
| Sum score |  | | | | | | | | | |
|  |  | | | | | | | | | |
| **Heart** | None  = 0 | | | Mild  = 1 | | Moderate  = 2 | | Severe  = 3 | |  |
| 1. Necrosis of cardiac muscle fiber |  | | |  | |  | |  | |  |
| 2. Infiltration of inflammatory cells |  | | |  | |  | |  | |  |
| 3. Dilatation of Henle space |  | | |  | |  | |  | |  |
| Sum score |  | | | | | | | | |  |

| The degree of **splenic** injury | **Score** |
| --- | --- |
| Normal morphology | 0 |
| Mild disorder of white pulp, characterized by local hyperplasia | 1 |
| Moderate disorder of white pulp, blurring of the boundary between white pulp and red pulp | 2 |
| High disorder of white pulp, almost no significant difference between white pulp and red pulp | 3 |
|  | Score= |
