# Supplementary material for: Intraperitoneal versus intranasal administration of lipopolysaccharide in causing sepsis severity in a murine model: a preliminary comparison
Source: Lab Anim Res. 2024 May 13;40:18. doi: 10.1186/s42826-024-00205-7 (PMC11089766; doi:10.1186/s42826-024-00205-7)
Supplement: Supplementary file 4 — Additional file 4. Histology of brain and heart from control and lipopolysaccharide (LPS) treated mice via intraperitoneal (I.P. 20_24 h & 10_96 h) or intranasal (I.N. 100_24 h & 20_96 h) routes at 24 h and 96 h stained with hematoxylin and eosin (H&E). Size bar = 50 μm. [file 42826_2024_205_MOESM4_ESM.docx]

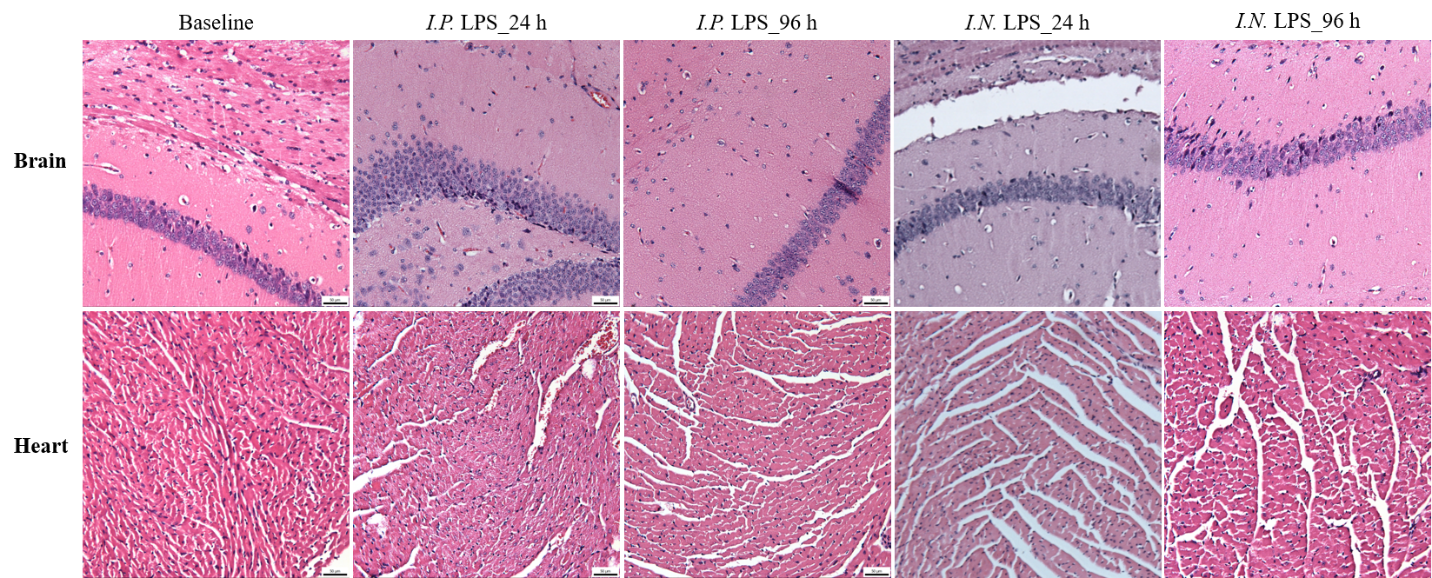


**Additional file 4** Histology of brain and heart from control and lipopolysaccharide (LPS) treated mice via intraperitoneal (*I.P.* 20_24 h&10_96 h) or intranasal (*I.N.* 100_24 h&20_96 h) routes at 24 h and 96 h stained with hematoxylin and eosin (H&E). Size bar = 50 µm.
