# Supplementary material for: Intraperitoneal versus intranasal administration of lipopolysaccharide in causing sepsis severity in a murine model: a preliminary comparison
Source: Lab Anim Res. 2024 May 13;40:18. doi: 10.1186/s42826-024-00205-7 (PMC11089766; doi:10.1186/s42826-024-00205-7)
Supplement: Supplementary file 5 — Additional file 5. Histology of lung, liver, spleen, kidney, brain and heart from 0.9% saline treated mice via intraperitoneal (I.P.) or intranasal (I.N.) routes at 96 h stained with haematoxylin and eosin (H&E). Size bar = 50 μm. [file 42826_2024_205_MOESM5_ESM.docx]

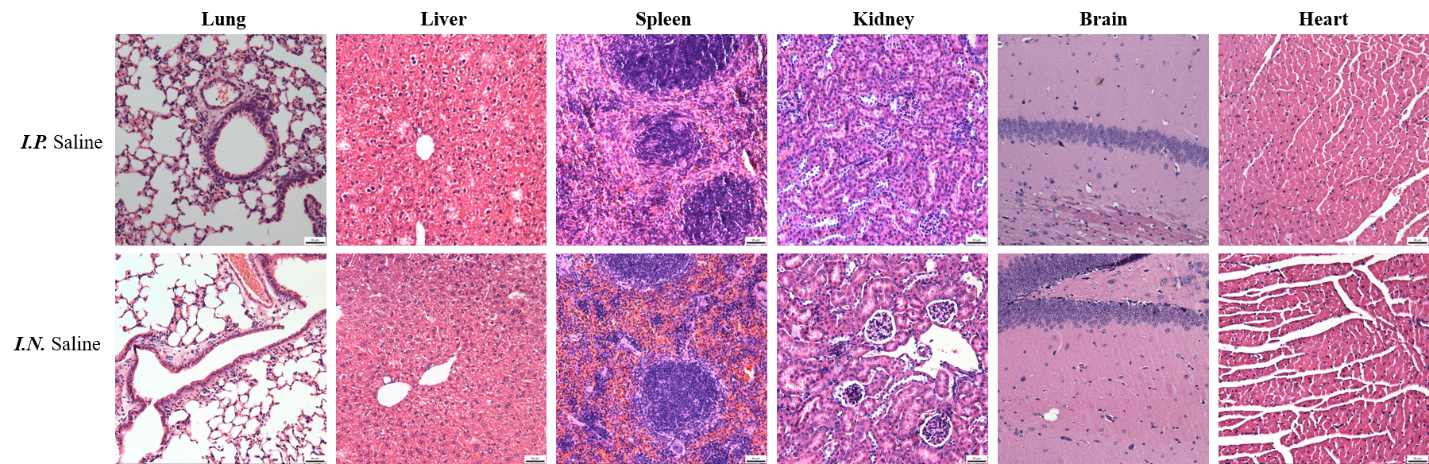


**Additional file 5** Histology of lung, liver, spleen, kidney, brain and heart from 0.9% saline treated mice via intraperitoneal (*I.P.*) or intranasal (*I.N.*) routes at 96 h stained with haematoxylin and eosin (H&E). Size bar = 50 µm.
