# Supplementary material for: Intraperitoneal versus intranasal administration of lipopolysaccharide in causing sepsis severity in a murine model: a preliminary comparison
Source: Lab Anim Res. 2024 May 13;40:18. doi: 10.1186/s42826-024-00205-7 (PMC11089766; doi:10.1186/s42826-024-00205-7)
Supplement: Supplementary file 6 — Additional file 6. Sequential Organ Failure Assessment (SOFA) score. [file 42826_2024_205_MOESM6_ESM.docx]

**Additional file 6** Sequential Organ Failure Assessment (SOFA) score.

| Score | 0 | 1 | 2 | 3 | 4 |
| --- | --- | --- | --- | --- | --- |
| Respiration PaO2/FiO2, mmHg | Normal | <400 | <300 | <200 (with respiratory support) | <100 (with respiratory support) |
| Coagulation Platelets x103/mm3 | Normal | <150 | <100 | <50 | <20 |
| Liver Bilirubin, mg/dL (μmol/l) | Normal | 1.2-1.9 (20-32) | 2.0-5.9 (33-101) | 6.0-11.9 (102-204) | >12.0 (<204) |
| Cardiovascular Hypotension | Normal | MAP<70 mmHg | Dopamine <5 or dobutamine (any dose) | Dopamine >5 or epinephrine <0.1 or norepinephrine <0.1 | Dopamine >15 or epinephrine >0.1 or norepinephrine >0.1 |
| Central Nervous System Glasgow Coma Score | Normal | 13-14 | 10-12 | 6-9 | <6 |
| Renal Creatinine, mg/dL (μmol/l) or Urine output | Normal | 1.2-1.9 (110-170) | 2.0-3.4 (171-299) | 3.5-4.9 (300-440) or <500 mL/day | >5.0 (>440) or <200 mL/day |
